# Correlation Between Viral Load of HBV in Chronic Hepatitis B Patients and Precore and Basal Core Promoter Mutations

**DOI:** 10.5812/hepatmon.7415

**Published:** 2013-02-18

**Authors:** Soad Ghabeshi, Zohreh Sharifi, Seyed Masoud Hosseini, Mahmood Mahmoodian Shooshtari

**Affiliations:** 1 Blood Transfusion Research Center, High Institute for Research and Education in Transfusion Medicine, Tehran, IR Iran; 2 Department of Microbiology, Faculty of Biological Sciences, Shahid Beheshti University, Tehran, IR Iran

**Keywords:** Mutations, Hepatitis B Virus, Viral Load

## Abstract

**Background:**

More than two billion people have been exposed to hepatitis B virus (HBV) worldwide. Furthermore, four hundred million of them are infected with chronic HBV infection. The predominant mutation of the precore region involves a G to A change at nucleotide1896, which creates a premature stop codon at codon 28. Two mutations of A1762T and G1764A are reported as the most prevalent mutations in the basal core promoter (BCP).

**Objectives:**

The purpose of this study was to investigate the relationship between mutations in precore (PC) and basal core promoter regions, and the viral load.

**Patients and Methods:**

Fifty serum samples from patients with hepatitis B were used. Levels of liver enzymes alanine aminotransferase (ALT) and aspartate aminotransferase (AST) were measured at the same time of serological markers of hepatitis B by ELISA. HBV-DNA was extracted from the sera, and then PCR performed on the HBV-DNA extracted with the use of specific primer of gene C. HBV viral load was determined by real-time PCR. The PC/ BCP mutations were determined by applying Line Probe Assay technique. The data were analyzed using SPSS software, version 20.

**Results:**

Only 82% of the patients were HBeAb positive and 76% of the patients had basal core/ precore mutations and mean viral load was 3/7 × 106 ± 9/7 × 105 IU/ml. Prevalence of mutations in the precore and basal core promoter regions were 46% and 30%, respectively.

**Conclusions:**

Our data indicated that there is a statistically significant relationship between HBV viral load and mutations in precore region (P < 0.05).

## 1. Background

Hepatitis B virus is a curricular and partially double-stranded DNA virus. Infection with HBV is recognized as the one of the most important reasons for viral hepatitis worldwide (1). It is possible that both host and viral factors have a role in severity of chronic HBV infection (1, 2). HBV genome has four open reading frames (ORFs), where negative and positive strands are in the same direction (in a clockwise direction). ORFC encodes c and e proteins. Core and precore ORF regions have the initiation codon (AUG) in a frame, which leads to synthesis of two similar proteins in their ends. Initiation of translation from the first AUG codon produces HBeAg protein and translation from the second initiation codon leads into synthesis of the nucleocapsid and core protein (3). A group of patients with permanent chronic hepatitis in the infection period have never been HBeAg positive, but they have been HBeAb positive and they are HBV-DNA positive. These patients suffer more severe liver disease compared to other patients infected with chronic hepatitis. Further studies on these patients have shown that high HBV viral load is accompanied by precore mutant (4, 5). The most prevalent mutation in the precore region belongs to a suppressing codon (codon 28), which leads into not producing HBeAg in the course of HBV infection. So, presence of HBeAb cannot be the reason for reduction in replication of the virus and low degree of clinical symptoms by the HBV in this group of patients (3, 6). This type of mutation is more prevalent in the countries of South Europe, Africa, the Mediterranean region, and the Far East Asia (3). Two mutations of A1762T and G1764A reported as the most prevalent mutations in the basal core region and described in different stages of HBV infection. Mutations in this region affect the expression of viral genes, replication, and probably viral pathogenesis (7). It has been proposed that changes in the secondary structure of the pregenomic RNA that give rise to the T1762/A1764 mutation may increase viral replication (8). HBV viral load in the patients infected with chronic hepatitis B depends on different factors such as individual immune response, environmental factors like alcohol consumption, and viral factors including genotype of the virus and presence of mutations in core and precore promoters (9).

## 2. Objectives

The purpose of this study was to determine the relationship between nucleotide changes in the basal core promoter and precore regions and viral load and serological markers.

## 3. Patients and Methods

### 3.1. Study Subjects

From May 2012 to October 2012, fifty HBsAg positive patients were referred to the medical laboratory of the Iranian Blood Transfusion Organization (IBTO).They signed an informed written consent and then filled out a questionnaire. The questionnaire included items on sex, age, marital status, past history of IV use of medication or drugs, family history of HBV, and blood transfusion. All the patients had chronic hepatitis B (defined as having the infection for more than six months and HBsAg positive). This study was approved by the medical ethics committee of the Iranian Blood Transfusion Organization (IBTO). Some of the patients received Lamivudin or interferon as a treatment for hepatitis B.

### 3.2. Laboratory Techniques

#### 3.2.1. Serological Markers

The serum samples were tested for the serological markers of hepatitis B including HBeAg, HBeAb, HBcAb, and HBsAg using the Enzyme Linked Immunosorbent Assay (ELISA) (DiaPro Diagnostic Bioprobes; Milano, Italy) according to the manufacturer’s instructions. (12)

#### 3.2.2. Biochemical Markers

Levels of liver enzymes alanine aminotransferase (ALT) and aspartate aminotransferase (AST) measured using Pars Azmoon kit (Tehran, Iran) based on the kit instruction.

### 3.3. HBV DNA Extraction

For carrying out PCR, extraction of HBV DNA was extracted from the sera of patients according to the manufacturer’s instructions using QIAmp DNA mini-extraction kit (Qiagen, Hilden, Germany).

### 3.4. HBV DNA Quantification

HBV viral load was assessed by real-time PCR using the Artus Light Cycler HBV DNA kit (Qiagen; Hilden, Germany); according to the manufacturer’s instructions and LightCycler 2.0 instrument Real-Time PCR (Roche, Germany).

### 3.5. Detection of Precore and Core Mutations

Detection of gene promoter polymorphisms in nucleotides 1762 and 1764 (BCP) and nucleotides 1896 HBV precore was performed by INNO-LiPA HBV Precore assay (Innogenetics; Ghent, Belgium). After purification of viral DNA from the patients’ samples, purified DNA was replicated in two stages using biotinylated primers. Then, the biotinylated DNA material generated from the basal core promoter and precore region was hybridized with specific oligonucleotide probes immobilized as parallel lines on membrane-based strips. Unhybridized DNA was then washed from the strip. Streptavidin labeled with alkaline phosphatase was added and bound to any biotinylated hybrid previously formed. Incubation with BCIP/NBT chromogen resulted in purple/brown precipitate.

### 3.6. Statistical Analysis

Results are expressed as mean ± SD. The data were analyzed SPSS software (version 20) by chi-square, Fisher’s test, Mann-Whitney’s, and independent samples t-tests. P values less than 0.05 were considered statistically significant.

## 4. Results

Fifty serum samples from patients with hepatitis B virus were used in this study. Of the patients, 68% were male (34 participants) and the patients’ mean age was 41.1 ± 21.05 year old. Serologic markers of HBeAg, HBsAg, HBcAb, and HBeAb were measured by ELISA. Mean viral load was detected as 3.7×106 ± 9.7×105 IU/ml. Nested PCR was carried out by the outer and nested primers. From the 50 samples, those having bands of 326- or 239-bp length were selected for the hybridization test. Among the 50 samples studied, 82% of positive PCR samples were HBeAb positive and 18% were HBeAg positive. Also, from the 50 samples studied, 24% did not have any mutations in the precore and basal core region while 46% had mutation from G to A in the precore region at nucleotide 1896 (codon 28) whereas 20% of them had mixed infection including two types of wild type and mutant viruses. Among the samples, 30% had dual mutations in the basal core promoter region, of which 93.3% were HBeAb positive. In the present study, we found two patterns of basal core promoter mutations included A1762A/G1764T and A1762T/G1764A. We did not observe the mutation pattern of A1762A/G1764A in the promoter core. Of the patients with the precore mutation, 26% had HBV viral load more than 105 IU/ml while only 6% of patients with high viral load had dual mutations in the basal core promoter regions. The results revealed that there is a statistically significant relationship between the HBV viral load and precore mutation (P < 0.05); while viral load and mutations in the basal core promoter region, were not significantly related (P < 0.3).There was no significant relationship between the sex of the patients and viral load (Table 1). Liver enzymes (ALT and AST) levels were also measured. Mean ALT in the patients with mutations in the basal core and precore regions was different from those without the mutation, (93.2 ± 22.4 vs. 40.53 ± 17.40, respectively). The mean AST values for the two groups were 66.9 ± 16.6 vs. 30.16 ± 13.94, respectively. Of the patients, 38% had received antiviral therapy. Among these patients, 57.8% did not have mutation in the PC and BCP. The results showed that there is a statistically significant relationship between receiving antiviral treatment and mutations in the core region (P = 0.05) (Table 2).

**Table 1.** Relationship between Mean ± SD Viral Loads and Risk Factors in Chronic Hepatitis B Patients

HBV Viral load Risk Factor, IU/ml	Mean ± SD	Minimum - Maximum	P value a
**Age, y (%)**			0.06
< 41 (56)	5 ×10^7^ (1 × 10^7^)	506-4 × 10^8^	
≥ 41 (44)	2×10^7^ (7 × 10^7^)	358-3 × 10^8^	
**Gender, %**			0.95
Male (68)	3.6 ×10^6^ (1 × 10^8^)	1950-4 × 10^8^	
Famale (32)	3.8 × 10^6^ (8.9 × 10^6^)	629-2 × 10^8^	

^a^chi-square analysis

**Table 2:** Age, Liver Enzyme Levels and Treatment in Patients with and Without PC and BCP Mutations

	With Pc and BCP Mutations	Without PC and BCP Mutations	P value
**Age, y, Mean ± SD**	41.09 ± 14.81	40.72 ± 18.84	0.25
**ALT, Mean ± SD**	93.2 ± 22.4	40.53 ± 17.40	0.001
**AST, Mean ± SD**	66.9 ± 16.6	30.16 ± 13.94	0.002
**Treatment, Yes/No, (%)**	16∕56	22/6	0.05

Abbreviations: ALT, alanine aminotransferase; AST, aspartate aminotransferase; BCP, basal core promoter; PC, precor

## 5. Discussion

The G1896A mutation in the precore region has been found in patients with HBeAg negative chronic hepatitis B. This mutation creates a stop codon that prevents translation of the PC protein and abolishes the production of HBeAg. These patients continue to synthesize HBV-DNA (1, 10). A1762T and G1764A double mutations can reduce HBeAg synthesis by inhibiting the transcription of the precore mRNA (11). Basal core mutations are more common in Asia and Pacific Ocean, where the C genotype is more prevalent. The precore mutations are more usual in the D genotype and are found more frequently in the Mediterranean region. However studies have shown that the spread of mutation in the precore region is not limited to this part of the world. In a study in 2009 on the commonest genotype, Milani et al. reported that the D genotype was detected in all the patients investigated (12). In the present study, the patients had genotype D which is consistent with the results of former studies. Patients with this genotype have more sever statuses of the infection and are less responsive to the interferon therapy in comparison with the patients having genotypes A or B (13-15). HBV-DNA level detection is a criterion for determining the state of infection, the risk of progression toward cirrhosis and HCC, identification of patients who need anti-viral therapy, determining response to therapy, and identifying emergence of drug resistance. Generally, serum HBV-DNA level significantly declines after eliminating of HBeAg. Some studies have confirmed the relationship between the viral load level and liver damage in patients negative for HBeAg whereas other studies have reported low-level viral load is not always an indication of improved conditions and in some patients it indicates advanced disease (9, 16, 17). In our study, 46% of the patients had mutations in the precore region, among which 82.6% were HBeAb positive. Mean viral load in these patients was high of the patients with the precore mutation, 26% had viral load above 105 copy/ml and ALT was in the upper normal range. This is consistent with what has been reported in former studies. Although these studies have shown that there is not a statistically significant relationship between mutations in this region and the HBV DNA levels, in most of these studies, high viral loads were detected in patients with the precore mutation (4, 5, 8). However we found a statistically significant relationship between having the precore mutation and high levels of HBV DNA. In an Iranian study, it was reported that prevalence rates of mutations in the precore and basal core regions were 31.8% and 38.6%, respectively. They reported that although there was no statistically significant difference between the patients with and without mutation in the precore and basal core regions in terms of age, gender, and liver enzyme levels (P > 0.05), liver enzyme levels in patients with mutations were higher than those in patients without mutations (10). Our results are in agreement with this local report which patients with mutations were older than those without mutations. It is believed that these patients have enough time for the PC region to replicate more (10). In the present study, there was no relationship between age and having the mutation. In our study, the prevalence of mutation was 30% (15 cases) in the basal core promoter region, among which 93.3% were HBeAb positive. Of the patients having mutations in the basal core promoter region, 18% had viral load less than 104 IU/ml. It has been reported that BCP mutation result in increased proliferation and reduced secretion of the virus in the blood that may increase the level of viral load in the liver and directly cause liver tissue damage (19). Our results are consistent with findings mentioned. There was no significant relationship between viral load and presence of mutations in the basal core promoter region (P < 0.3). This is while some studies revealed that there is a significant relationship between mutations in the basal core region on the one hand and viral load and liver damage on the other hand (18-20). This discordance between our results and such reports may be due to the low number of samples in our study. Among the patients, 24% lacked any mutations in the precore and BCP region that 8% of them had viral load above 105 IU/ml, with ALT levels within the normal range. These patients might be infected during childhood and be in the immune tolerance status (19). In a study, it was suggested that antiviral treatments are more potent in PC or BCP mutants than in wild-type virus. (21). Our results are compatible with these findings. Also, it has been reported that during the course of antiviral therapy, reversions from PC/BCP mutants to wild type HBV may occur (21, 22). In summary, in the current study, there is a relationship between mutation in the precore region and high viral load, which can involve in severity of liver damage. Our study showed that the replication activity of the basal core promoter mutants has little effect on the viral levels in patients and viral load may be a major determinant of the liver damage severity in chronic hepatitis B patients. Moreover, our results suggested that HBV DNA level should be carefully monitored after HBeAg clearance. Mutations in precore region of HBV are associated with high viral load. High HBV-DNA levels in HBeAb positive patients requiring follow-up and antiviral therapies.
